# Imaging of X-Ray-Excited Emissions from Quantum Dots and Biological Tissue in Whole Mouse

**DOI:** 10.1038/s41598-019-55769-5

**Published:** 2019-12-16

**Authors:** Sean G. Ryan, Matthew N. Butler, Segun S. Adeyemi, Tammy Kalber, P. Stephen Patrick, May Zaw Thin, Ian F. Harrison, Daniel J. Stuckey, Martin Pule, Mark F. Lythgoe

**Affiliations:** 1 School of Physics, Astronomy and Mathematics, University of Hertfordshire, College Lane, Hatfield, AL10 9AB UK; 2 School of Health and Social Work, University of Hertfordshire, College Lane, Hatfield, AL10 9AB UK; 3 Centre for Advanced Biomedical Imaging, University College London, 72 Huntley Street, London, WC1E 6DD UK; 4 Cancer Institute, University College London, 72 Huntley Street, London, WC1E 6DD UK

**Keywords:** Diagnostic markers, Preclinical research

## Abstract

Optical imaging in clinical and preclinical settings can provide a wealth of biological information, particularly when coupled with targetted nanoparticles, but optical scattering and absorption limit the depth and resolution in both animal and human subjects. Two new hybrid approaches are presented, using the penetrating power of X-rays to increase the depth of optical imaging. Foremost, we demonstrate the excitation by X-rays of quantum-dots (QD) emitting in the near-infrared (NIR), using a clinical X-ray system to map the distribution of QDs at depth in whole mouse. We elicit a clear, spatially-resolved NIR signal from deep organs (brain, liver and kidney) with short (1 second) exposures and tolerable radiation doses that will permit future *in vivo* applications. Furthermore, X-ray-excited endogenous emission is also detected from whole mouse. The use of keV X-rays to excite emission from QDs and tissue represent novel biomedical imaging technologies, and exploit emerging QDs as optical probes for spatial-temporal molecular imaging at greater depth than previously possible.

## Introduction

Over the last four decades, biomedical imaging has revolutionised preclinical and clinical medicine with highly detailed structural and functional scanning. Optical imaging, such as fluorescence or bioluminescence imaging, can provide a wealth of biological information using wide range of small molecular probes or genetically encodeable reporter proteins. However, optical scattering fundamentally limits the penetration depth of both the exciting and the emitted radiation, and limits the achievable spatial resolution. X-ray excitation of NIR-emitting probes offers the prospect of overcoming these limitations, particularly when applied tomographically. In this study we demonstrate the potential of X-ray-excited nanoparticle imaging of quantum dots to produce a NIR signal suitable for biomedical imaging in deeper tissue.

Recent developments in imaging and biomedical research have enabled the use of nanoparticles to help target physiological features or functional sites of interest, for diagnostic^1–4^ and therapeutic^5^ purposes. As such, there is great interest in tracking such nanoparticles, for example in oncological targetting^2,4,5^, but using external sources of UV/optical/NIR excitation for *in vivo* imaging is challenging because absorption and scattering result in limited penetration depth and resolution, and can create confounding background autofluorescence from tissue chromophores. Absorption and scattering similarly limit the depth at which photodynamic therapy (PDT) can be conducted. One approach aimed at overcoming these limitations uses penetrating X-rays to excite radio-luminescent nanoparticles in such a way that optical/near-infrared (NIR) light emitted can be detected externally. These techniques include X-ray luminescence computed tomography (XLCT)^6–9^, also known as X-ray radio-luminescence CT and X-ray scintillation CT, where the scintillating chemical is often the rare-earth phosphor Eu (e.g. Gd_2_O_2_S:Eu, BaYF_5_:Eu^3+^, Gd_2_O_3_:Eu^3+^)^9,10^, and X-ray luminescent optical tomography (XLOT) utilising Gd_2_O_2_S:Eu^3+^^11^. (In contrast, X-ray fluorescence CT (XFCT) emits at X-ray energies^6–8^). However, it is important to investigate alternative nanoparticles, particularly those that are physically smaller, brighter, more biocompatible, and offer deep-tissue compatible emission wavelengths further into the NIR than the Eu^3+^ ~610 nm line, in short, that are better suited to biomedical imaging. Quantum dots (QDs) constitute one potential such alternative that has seen increased utilisation for imaging and drug delivery in recent years^2,5,12^.

The high quantum yields and size-dependent (tuneable) emission of QDs make them appealing as optical probes for biological imaging. They are smaller than some luminescent phosphors, being 1–10 nm diameter nanocrystals of semiconductors, typically pairings of periodic table groups II-VI, e.g. CdSe and CdTe, or III-V, e.g. GaN. ZnS/CdSe QDs have been used at 606–655 nm to image tissue at shallow depths^13^. NIR-emitting QDs are of particular interest because of the low-absorption window for haemoglobin and water between 700 and 900 nm^1^, and reduced scattering at longer wavelengths^14^. NIR light thus takes a less tortuous path through deep biological tissue. The scattering coefficient in this waveband is higher than the absorption coefficient, so scattering, albeit strongly forward-peaked (at least in the case of human breast tissue^14^), dominates. Being smaller than some nanophosphors, QDs can move from the vascular system into tissue, they are configurable in a greater range of emission wavelengths, are more resistant to photo-bleaching, can be functionalised, capped or manufactured from materials free of Cd, Hg and Pb to render them non-toxic^2,12,15,16^ and can have shorter de-excitation times than excited fluorescent dyes^17^.

Reported attempts to excite QDs with X-rays have met with limited success. The excitation of dry CdSe/ZnS core-shell QDs (λ_emission_ = 510 nm) embedded in porous glass, using 59 keV γ-rays (1 μCi ^241^Am source) yielded a positive result, but only after a lengthy 3 day integration time to achieve ~1600 counts^18^. Less positively, X-ray luminescence that was achieved for CdTe nanoparticles encapsulated at trace compositions (0.3–0.6% by mass) in a BaFBr:Eu^2+^ phosphor, using 50 kV_p_ X-rays (1 mA, tungsten anode), was found to cease for non-encapsulated CdTe^19^ and no X-ray luminescence was found in the CdTe/BaFBr composite without the Eu^2+^ ion, implying that the Eu^2+^ ion was crucial in the conversion of X-ray excitation to emission in that system. Even five years later, Kang *et al*.^17^ remarked that the “x-ray excitation mechanism in quantum dots is still not clear”.

Nevertheless, we pursued a series of experiments to achieve and quantify X-ray excited NIR emission from, initially, core-type CdTe QDs in aqueous suspension in phantom and mouse models, using a standard clinical X-ray system at energies between 20 and 120 keV. We succeeded in generating high image contrast from X-ray-excited luminescence of CdTe QDs, and we report the results here. We also report X-ray-excited optical fluorescence of mouse tissue without priming by QDs, presenting a new diagnostic of future potential application.

## Results

**Figure 1:**
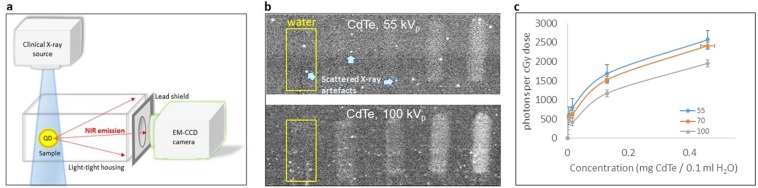
Excitation of NIR emission from quantum dots by X-ray excitation. **(a**) The excitation and imaging system uses a clinical diagnostic X-ray source operating at keV energies, irradiating a sample within a custom-made light-tight housing. NIR emission from QDs in the sample is detected using an off-the-shelf EM-CCD and lens, the latter protruding into the light-tight box through a lead shield either directly (as illustrated) or via a 45° fold mirror to improve the exclusion of scattered X-rays. (**b**) NIR images of five syringes, arranged side-by-side, containing 0.1 ml aqueous suspensions of, from left to right, 0 (control), 0.005, 0.016, 0.13 and 0.46 mg of CdTe, irradiated by X-rays at (top) 55 kV_p_ and (bottom) 100 kV_p_. In all cases, the first syringe containing the water control (yellow box) has no visible emission. The 100 kV_p_ image is the average of four 1 second exposures with maximum-pixel rejection (to minimise scattered X-ray contamination; examples of artefacts indicated). (**c**) Variation of detected NIR emission per cGy dose with CdTe concentration and kV_p_ setting, over rectangular regions of interest (ROIs) for each syringe. The typical uncertainty (1*σ*) is ± 92 photons per cGy at 70 and 100 kV_p_, and ± 226 photons per cGy at 55 kV_p_ (where only one frame was recorded – Table 1). A 5% uncertainty in concentration is assumed.

The X-ray excitation and NIR imaging system is shown schematically in Fig. 1(a). It consists of a keV clinical radiographic X-ray source positioned over a sample located in a standalone light-tight housing. The cone of X-rays emitted from the source enters the housing through its X-ray-transmitting shell and passes directly through the sample. An off-the-shelf EM-CCD camera, the lens of which protrudes into the housing from behind a lead shield, records X-ray-excited NIR emission from the QDs.

### NIR emission from QDs in phantom (syringe) imaging

Five 2.9 mm internal-diameter syringes (0.3 ml insulin syringes) were loaded with 0.1 ml aqueous suspensions of 715 nm-emitting CdTe QDs, containing 0, 0.005, 0.016, 0.13 and 0.46 mg of CdTe. A sequence of images was obtained for 55, 70 and 100 kV_p_ irradiation (Table 1). Exposures were also made with the X-rays off, to provide “background” images with no emission; the difference images showing X-ray excitation are in Fig. 1b and are quantified in Table 1. The NIR emission increases, though non-linearly, with CdTe concentration (Fig. 1c), confirming that the emission arises from the QDs. The 55 kV_p_ setting has the lowest counts but this was achieved at significantly lower dose (see dose-area product (DAP) in Table 1) than the 70 kV_p_ and 100 kV_p_ exposures, and represents higher emission per unit dose (Fig. 1c), as expected since X-ray absorption is higher at lower keV. Additionally, at 55 kVp, the CCD is more effectively shielded by a given thickness of lead, reducing the noise on the CCD from scattered X-rays.

**Table 1:** Exposure parameters, measured dose-area product (DAP) and background-subtracted photon count per second, for four 0.1 ml CdTe suspensions in syringes, based on rectangular ROIs covering each 0.1 ml suspension in the images.

kV_p_	Beam current	Exposure time	DAP	Excess signal in ROI (relative to water control) for four CdTe concentrations:			
				**0.005** **mg**	**0.016** **mg**	**0.13** **mg**	**0.46** **mg**
**[kV]**	**[mA]**	**[ms]**	**[cGy cm**^**2**^**]**	**[photons s**^**−1**^**]**			
55	500	1 × 1000	140	2180	3009	6283	9591
70	500	3 × 1000	230	3606	4112	9956	15767
100	250	4 × 1000	230	3357	2702	7659	12749

See also Fig. 1(b,c).

The quantum yield from QDs can be enhanced by adopting core-shell or alloyed structures (to passivate surface trap states) rather than simple core QDs^20^. A suspension of Cd-free ZnCuInS/ZnS core-shell QDs with an emission wavelength of 700 nm likewise gave rise to bright NIR emission under X-ray excitation, confirming that X-ray excitation of QDs is not confined to CdTe. Unexpectedly, we found that the ZnCuInS/ZnS QDs are not just luminescent but also phosphorescent under X-ray excitation, so for the present paper we focus solely on X-ray excitation of CdTe QDs.

### NIR emission from QDs in subcutaneous mouse imaging

**Figure 2:**
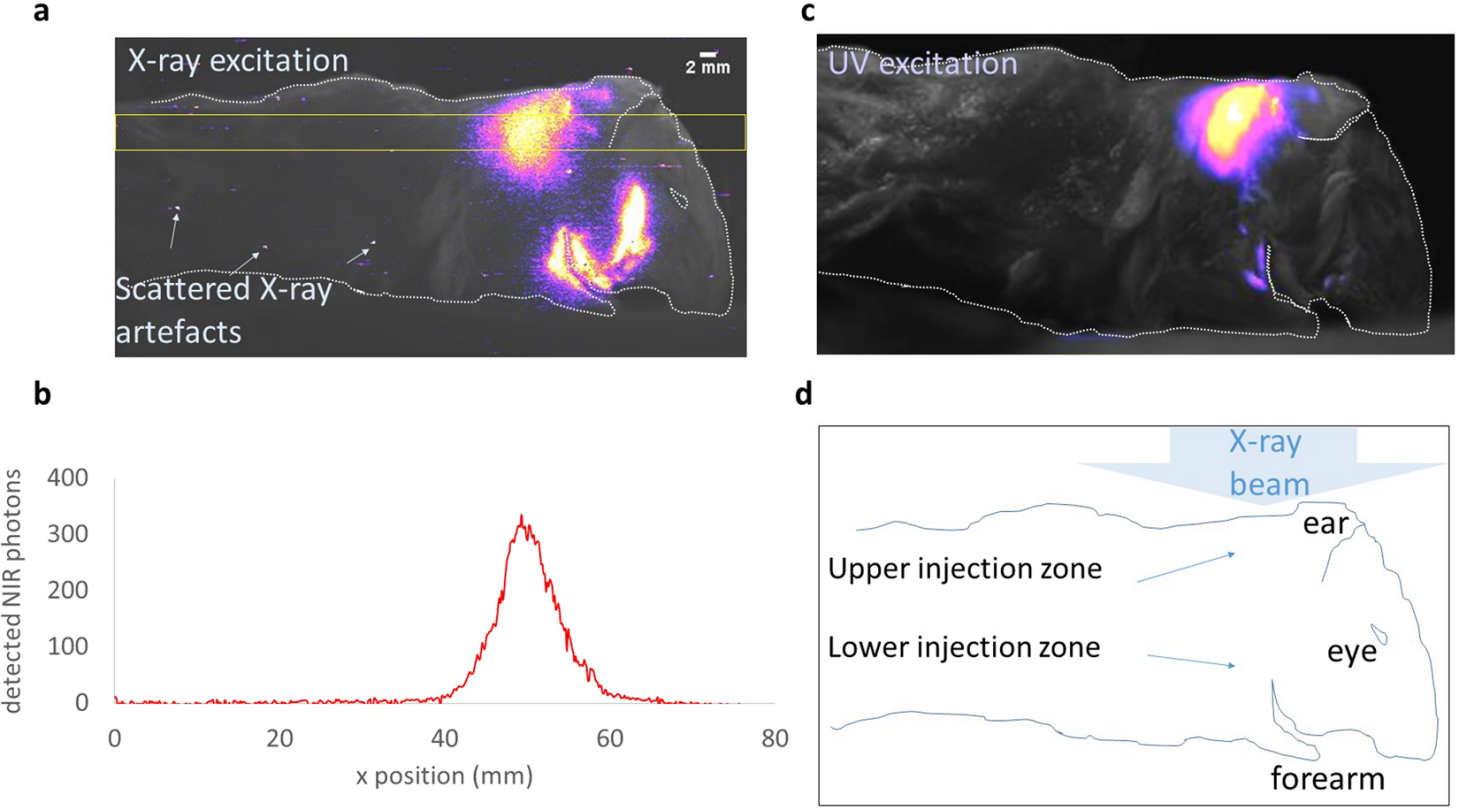
X-ray-excited NIR emission from whole mouse cadaver, recorded on EM-CCD. (**a**) (Greyscale) Ambient-light NIR image of mouse cadaver which has been injected at two points with CdTe QD suspensions having λ_emission_ = 715 nm, and (colour) superimposed detected 715 nm NIR emission excited by X-rays at 55 kV_p_. (A small number of pin-point artefacts exist due to scattered X-rays). The X-ray source is overhead, at 50 cm SSD. The yellow lines mark the boundary of an ROI examined in (**b**). (**b**) Cross-section showing the number of X-ray-excited NIR photons detected over the 26 rows in the ROI of the image in (**a**). (**c**) UV-excited QD emission (colour) superimposed on ambient-light image (greyscale). The UV source is directly overhead, as for the X-ray source that excited image (**a**), but due to UV absorption by the whole-mouse tissue and bone, no UV excitation of the lower injection zone is achieved, whereas the 55 kV_p_ X-rays readily penetrate this material and produce the QD excitation seen in (**a**). The images confirm that the X-ray-indicated distribution of QDs matches that inferred by conventional UV excitation for the upper injection zone. (**d**) Schematic to aid the interpretation of images (**a**,**c**), showing the regions of the two shallow QD injections, plus the eye, ear (folded forward), and forearm (the paw is tucked under and not visible).

We injected two sites subcutaneously in a mouse cadaver, one (lower) in the shoulder (0.26 mg CdTe) and a further (upper) near the back of the neck (0.46 mg CdTe) (Fig. 2). X-ray irradiation was set at 55 kV_p_ and 500 mA for 1 second, at 50 cm source-to-subject distance (SSD). The collimator exposed a rectangular region covering the full head and neck. Figure 2(a) shows one a typical image, revealing unambiguous X-ray excited QD emission (shown coloured) at two sites corresponding to the two injections, superimposed on a (greyscale) optically-illuminated image for registration purposes. Figure 2a is one frame from a series of 11 viewing angles, for which a montage and a stop-motion video is available (see Data Availability for URL). Figure 2b shows the cross-section through a 26-row ROI through the upper injection point of the X-ray excited image, and reveals that several hundred photons were detected per EM-CCD column within that ROI. The detection is therefore unambiguous, with a signal-to-noise ratio per column up to 18 (based on Poisson statistics for photon counting).

Verification that the emission seen in Fig. 2a is in fact due to X-ray excitation of the QDs, and that the X-ray-indicated distribution is reliable, is provided in Fig. 2c. In this image of the same mouse, acquired with the same camera, lens and NIR filter, the QDs have been excited conventionally using UV light (Hg emission lamp + BG39 filter). The UV lamp was positioned directly overhead, as for X-ray excitation. The X-ray-indicated distribution of the QDs in the region of the back of the neck (Fig. 2a) matches extremely well the distribution revealed by UV excitation (Fig. 2c), but because of the absorption of the UV rays by the 1-2 cm path-length through the whole-mouse tissue and bone, UV light is prevented from reaching and exciting the lower zone of QDs. In contrast, the X-rays readily penetrate this thickness of tissue and bone, indicating the increased depth of penetration of the X-rays and their excitation of QDs (Fig. 2a). Figure 2a thus confirms the ability of 55 kV_p_ X-rays to excite QDs after penetrating 1-2 cm of overlying tissue. In the next subsection we demonstrate that not only is excitation achieved at depth, but that NIR light emitted at depth can be detected at the surface.

### NIR emission from QDs in whole body, deep tissue mouse imaging

Following the successful detection of subcutaneously-injected QDs, we sought confirmation that we could also excite and detect them at depth in whole mouse. Three injections, each containing 0.5 mg CdTe in 2 μl aqueous suspension, were made in a nude-mouse cadaver: one in the left striatum of the brain, one in the liver and one in the left kidney. The first was performed with the head of the mouse immobilised in a stereotaxic frame, while the second two were conducted under ultrasound guidance, to ensure the QDs were injected where intended.

**Figure 3:**
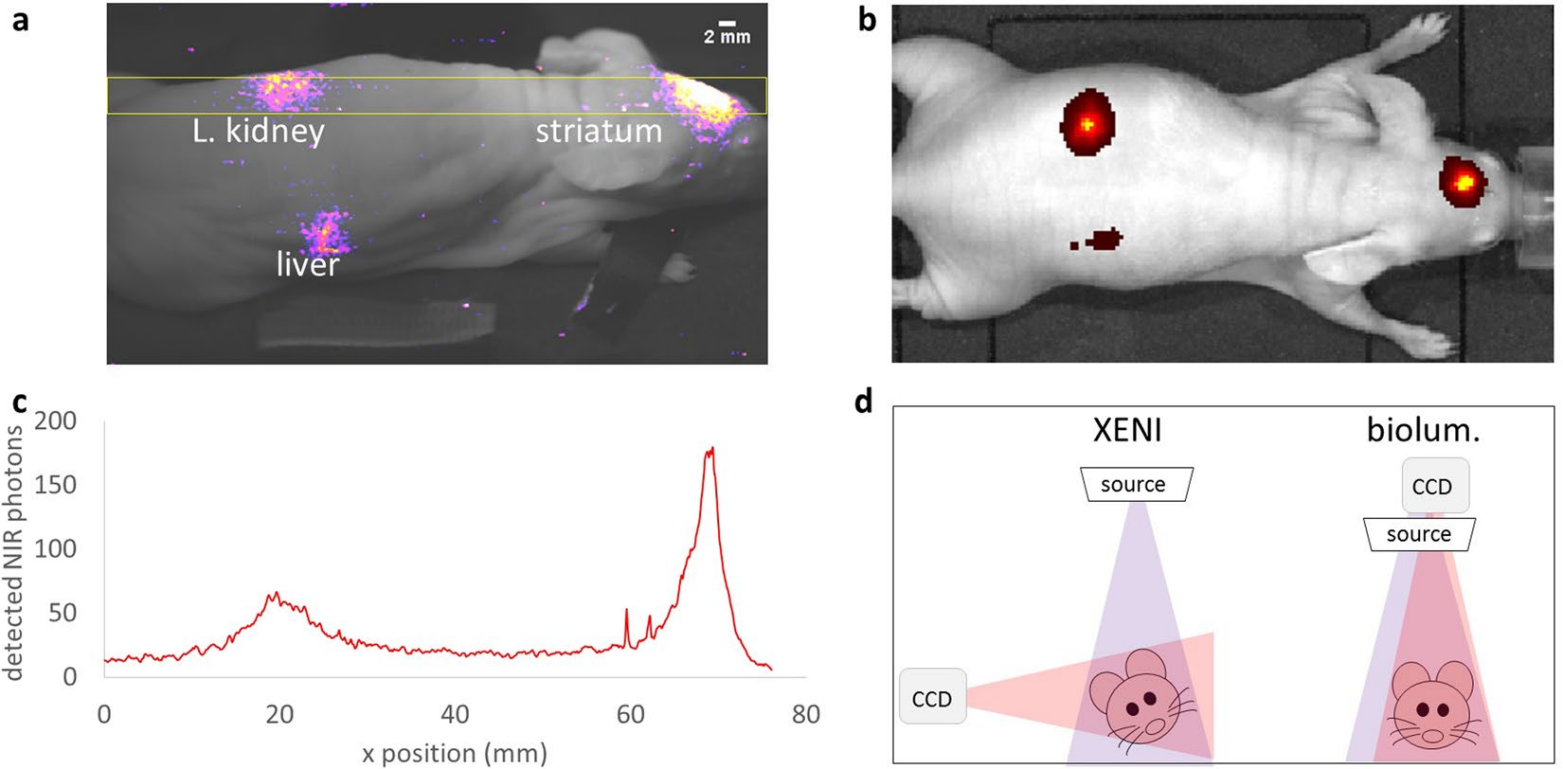
NIR emission from CdTe QDs injected into left striatum, liver and left kidney of nude mouse cadaver. (**a**) (colour) X-ray-excited NIR image recorded with our system, superimposed on (greyscale) ambient-light image. (**b**) optically-excited (500–550 nm) NIR image of QD distribution recorded with industry–standard bioluminescence and fluorescence detection system. The similarities between the two images validate that our X-ray-excited nanoparticle imaging technique is capable of exciting detectable NIR emission from deeply-embedded QDs. (**c**) Signal profile along the 26 row ROI shown (yellow) in (**a**). (**d**) Schematic of different geometries for imaging with our X-ray-excited nanoparticle imaging technique (XENI) and standard bioluminescence and fluorescence (“biolum.”) imaging. As currently configured, the X-ray beam enters at 90° to the imaging direction, whereas standard bioluminescence and fluorescence imaging has both the excitation illumination and CCD directly above the sample. The X-ray excited QD detection of the liver is achieved despite the thickness of tissue the X-rays must first pass through, whereas the bioluminescence and fluorescence geometry is optimised for more superficial fluorescence near the upper surface.

The mouse cadaver was then imaged as above, with X-ray irradiation at 55 kV_p_ and 500 mA for 1 second, and 50 cm source-to-subject distance (SSD). The resulting images (e.g. Figure 3a) show the three injection sites very clearly, indicating that X-rays reach these deeper locations and that the 715 nm radiation emitted by these deeper QDs is visible at the skin surface.

Verification of the QD distribution in the mouse was obtained by also obtaining images from an IVIS Lumina (Perkin Elmer) under DsRed excitation (500–550 nm) and Cy5.5 emission (690–770 nm). Figure 3(b) confirms that the distribution of QDs identified by X-ray excitation matches that inferred from a state-of-the-art, industry-standard fluorescent imaging system. Our contention here is not that X-ray excited nanoparticle imaging should replace state-of-the-art optical fluorescence imaging, but rather provides an alternative imaging system for organs at depth in whole mouse, through X-ray excitation of QDs. Future applications of the technique will be described in the discussion.

The signal profiles of these microlitre QD volumes is determined by multiple scattering events which photons experience as they traverse deep tissue. The typical path-length between NIR scattering events is only around 30 μm^14^, but as the scattering is strongly forward-peaked^14^, the emergent NIR photons nevertheless retain valuable positional information. The signal profile (Fig. 3c) along a 26 row ROI of the X-ray excited image (shown yellow in Fig. 3(a)) shows that images of the kidney and brain QD sites have FWHM values 6–8 mm which, for the photon counts *N* in Fig. 3(c), indicates the centroid of each distribution can be located to an accuracy better than 1 mm (σ/*N*^1/2^).

The potential of X-ray excited nanoparticle imaging is further illustrated by the clear detection of the liver in the X-ray-excited view of Fig. 3(a), even though the X-rays pass through 2 cm of overlying tissue to reach the liver. In contrast, the attenuation of optical excitation radiation is evident from a comparison of the fluorescence images (Fig. 3(b,d)).

### Non-QD endogenous emission from biological tissue following X-ray-excitation

Whole-mouse images taken prior to shaving and depilation, without filtering and thus sensitive to the full spectral range of the EM-CCD, show an extended region of emission arising from the mouse cadaver. The detections, achieved at three different kV_p_ settings, indicate that X-ray-excited endogenous radioluminescence it emitted from the natural biological tissue of the mouse, distinct from and fainter than the NIR QDs emission. The detection of X-ray-induced endogenous radioluminescence is, to our knowledge, a novel finding arising in this study.

**Figure 4:**
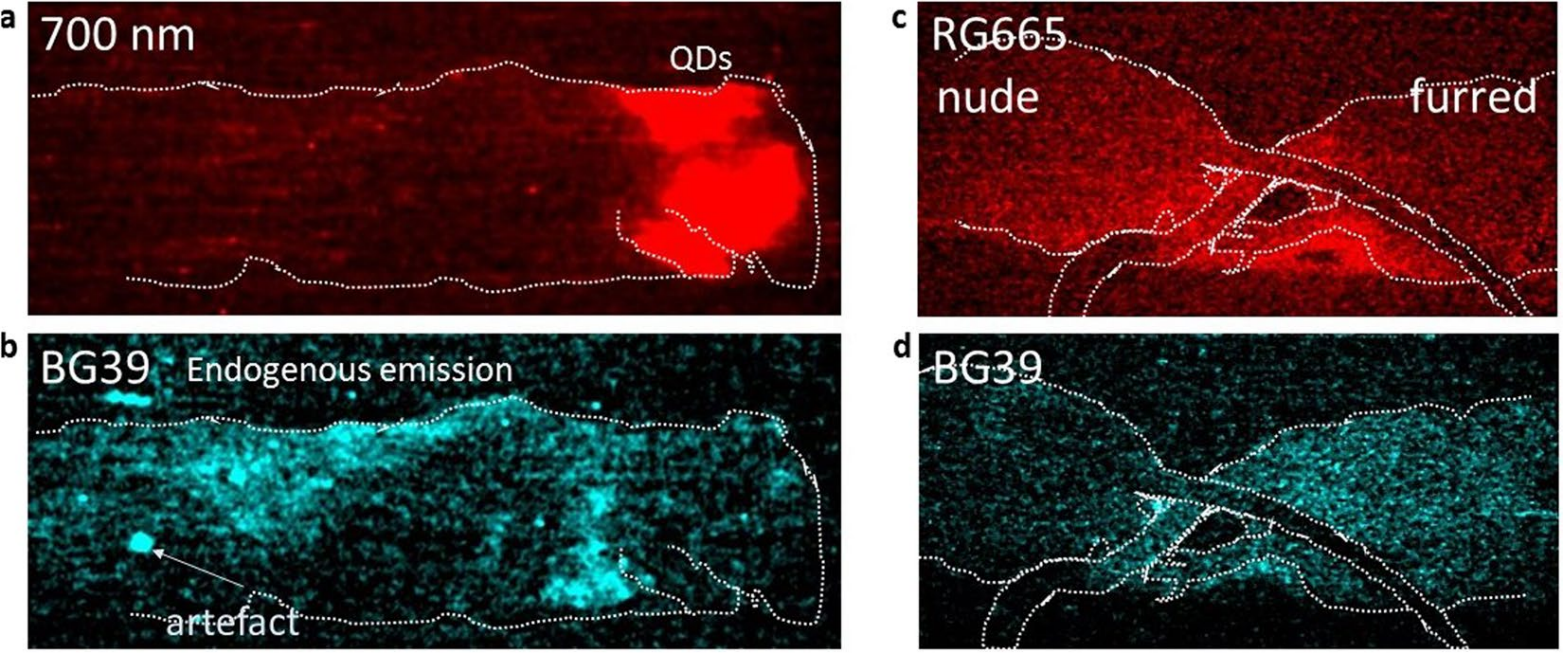
X-ray-excited endogenous radioluminescence from mouse cadavers with and without QDs. X-ray excitation at 55 kV_p_. The intensity scale has been set to accentuate the faint end of the intensity scale. (**a,b**) Partially depilated, furred mouse with QD injection, orientation as for Fig. 2. (**a**) (red) 700 nm narrowband (FWHM 70 nm) filter accentuating 715 nm QD emission. (**b**) (cyan) Broadband BG39 filter to accentuate endogenous emission at wavelengths < 600 nm. The distribution of endogenous radioluminescence in (**b**) indicates it arises mostly from the fur. (The bright spot in the leftmost quarter of image (**b**) is a detector artefact). (**c,d**) Rump of (left) nude and (right) furred mouse without QDs. (**c**) Broadband red (RG665) image. (**d**) Broadband blue (BG39) image showing that the furred mouse emits more strongly than the nude mouse in the 320–660 nm band. The curved dark band is the silhouette of the nude mouse tail (not irradiated) blocking the emission from the furred mouse.

Expecting the endogenous radio-luminescent properties might depend on tissue type, we used a depilating cream (Veet) to remove much of the fur from the upper torso and neck, but left the rump unchanged. Figure 4(a,b) shows two images at 55kV_p_ taken with the full body exposed to X-rays, orientated as in Fig. 2. Figure 4(a) was taken through a narrow band red filter centred on 700 nm, showing the concentration of the QD emission around the neck and head, whereas Fig. 4(b) is taken through a broadband BG39 (320–660 nm) blue-coloured filter showing that the endogenous emission in this case arises more strongly from the furred regions of the mouse.

To further characterise the endogenous radio luminescence, we imaged simultaneously two additional whole mice cadavers, one furred and one nude, which had not been injected with QDs. These images, shown in Fig. 4(c,d), demonstrate more clearly the endogenous emission free of a QD contribution. Both cadavers emit clearly under 55 kV_p_ excitation. Imaging through (Fig. 4(c)) broadband red (RG665) and (Fig. 4(d)) broadband blue (BG39) filters indicates that the furred mouse emits more strongly than the nude mouse in the 320–660 nm band. This endogenous radio luminescence is therefore clearly tissue dependent, and offers the potential for a further diagnostic imaging strand in X-ray excited imaging, albeit with a fainter signal than for QD emission.

Further imaging through narrower bandpasses within the BG39 range showed no detectable emission in the green 515–590 nm band, but a detectable signal from the furred mouse in the violet 380–430 nm and blue 425–470 nm ranges. The violet and blue ranges coincide with the fluorescence of collagen (~380 nm), elastin (~410 nm) and NADH (~460 nm), while the green range where we detect no signal coincides with flavin (~525 nm)^21^. While we cannot definitely identify the observed emission with collagen, elastin and NADH, these are possible identifications for further exploration. Differences in the optical fluorescence of normal and cancerous tissues have been noted elsewhere^21^, and are reasonable expectations for the radioluminescence as well.

We note finally that radioluminescence also serves as a potential confounder of QD emission, and underscores the importance of matching the optical filtering during image acquisition to the emission spectrum of the QDs. It may also present one cautionary counter-example to statements that autofluorescence is not a confounder of XLCT^9^.

### Operational efficiency

We define the operational efficiency of the QDs as the NIR energy radiated into 4π steradians divided by the X-ray energy deposited in the suspension (including the water). From the number of photons detected from the 55 kVp –irradiated syringe, and the corresponding X-ray dose values, we infer operational efficiencies around 1 × 10^−6^ for CdTe concentrations 0.1 mg per 0.1 ml. At lower concentrations the efficiency falls, as the X-ray dose is still delivered (predominantly to the water) but, with fewer QDs, there is less NIR emission.

This definition of the operational efficiency is less helpful for the mouse, since the quantity of fluid capable of absorbing X-rays is not simply the bolus size, as the bolus will be in close proximity to other fluids and tissue. We therefore quote instead the imaging parameters for Fig. 2. For a dose of 3.7 cGy (55 kVp, 500 mAs, 50 cm SSD), we detected 41600 NIR photons s^−1^ from the upper, subcutaneous injection site containing 0.46 mg CdTe in suspension. There is clearly scope to reduce the X-ray dose and/or QD concentration, and to image at greater depth, while retaining significant photon counts.

## Discussion

Our measurements have shown that X-ray excitation of NIR emission from CdTe core-type QDs and ZnCuInS/ZnS core-shell QDs can be achieved using clinically-relevant X-ray energies (20–120 keV) and doses (cGy), and that the emission is intense enough to be imaged in 1 sec using a sensitive EM-CCD detector. This result ushers in a new, practical and convenient biomedical imaging technology that benefits from a gain in the depth of imaging due to the penetrating power of X-rays compared to UV/optical exciters of fluorescence, yielding NIR emission that experiences low absorption by water and blood^1^. We have demonstrated that the X-ray-excited NIR emission can be readily detected from QDs deposited in liver, kidney and brain in whole mouse. This is a significant step towards the practical application of QD based reporters at depth *in vivo*. We are confident of developing preclinical and potentially clinical applications of this new technique.

Because of the high penetrating power of keV X-rays, the positioning of the X-ray source is less important than optimising the emergence pathway of the QD NIR emission and its capture on the NIR detector (in this case an EM-CCD). The X-ray penetrating power also gives this technique different capabilities to methods based on UV- and visible-light excitation such as 3D-fluorescence and photo acoustic imaging, and techniques where tissue clearance is necessitated, e.g. in mapping vascular networks^22^. The X-ray beam shape can be controlled by adjustments to the collimator, to restrict the excitation dose to the region of tissues of interest. Combining the targeted delivery of QDs *in vivo* and *in vitro*, and their selective excitation by an even more finely collimated X-ray beam will allow higher resolution imaging than that demonstrated here. Indeed, future tomographic approaches are envisaged, where knowledge of the collimated X-ray trajectory and the distribution of the emergent NIR light will permit 3D modelling of the QD locations with sub-mm resolution. Tomographic applications will possess similar characteristics to nanophosphor modalities such as XLCT and XLOT, but with additional capabilities and sensitivity associated with the small size of QDs and their greater mobility in biological systems. As our current (non-tomographic) imaging is relatively insensitive to the X-ray path, the technique may also benefit *in vivo* preclinical studies where anaesthesia is a potential confounding factor^23^, affecting as it does animal metabolism. We will seek to explore *in-vivo*, awake small-animal imaging using X-ray excitation of targeted QDs, to ascertain whether we can achieve internal morphological and ultimately functional imaging via this method.

Our detection of radioluminescence from QDs in 1 second exposures contrasts with non-detections, or detections requiring multi-day exposure times, reported for *dry* QD samples elsewhere^18,19^. We note that our results were obtained for QDs in *liquid suspension*, even at low concentrations < 0.5 mg per ml in the case of Fig. 1. The greater dispersal of QDs in transparent liquid suspensions (water for CdTe; toluene for ZnCuInS/ZnS) and their resultant NIR transparency may account for our success compared to previous dry experiments. Relatedly, it is important to note that more X-ray energy is deposited initially in the liquid of the suspension than in the QDs; the mass of water in the syringe suspension (100 mg) exceeded the mass of CdTe (<0.5 mg) by a factor of at least 200, which is much larger than the ratio of X-ray absorption coefficients^24^. Some of the X-ray energy deposited initially in the liquid will be available to the QDs, as at ~55 keV the dominant mechanism of X-ray energy deposition is the photoelectric effect, where the X-ray is destroyed and a photoelectron is liberated, initially with a kinetic energy up to tens of keV. Secondary electrons and additional high-energy photons are generated, producing further ionisations and excitations in their passage through the material until the original X-ray energy is dissipated. The small size of the nanoparticle (~5 nm) compared to the relatively long mean-free path for energy deposition by secondary electrons (~1 μm) means that only a small fraction of the X-ray energy deposited *within* a QD will remain there^25^, but conversely, photoelectrons, secondary electrons and photons generated by X-rays absorbed in the liquid, where the majority of X-rays are absorbed, will also transfer energy to, and excite, the QDs. By the same mechanism, we see the excitation of endogenous emission in Fig. 4, and would expect to be able to excite administered fluorescent dyes and other materials with luminescent centres the same way though we have not sought to verify that.

Radiation doses for 55 kV_p_ were in the range 3-4 cGy; these are a factor of ten larger than is typical for diagnostic spinal X-rays^26^, but lie well within the 0–2 Gy range for acute skin dose in humans within which no adverse observable effects are expected^27^. For preclinical applications involving animals with naturally short lifetimes, the long-term risks normally associated with exposure of humans to ionising radiation may be moot.

Other core-shell or multi-layer QDs^20^ offer further opportunities to improve the luminous efficiency (quantum yield) and avoid potential side-effects associated with CdTe^2^. Once capped with amphiphilic poly(acrylic acid) polymer, even Cd-containing ZnS/CdSe QDs were found^13^ to be maintained *in vivo* in mouse for 4 months without causing detectable necrosis or undergoing breakdown. This provides encouragement that even current Cd-containing QDs can find application in preclinical imaging, and that with the further development of multi-layer and Cd-free QDs^2,20^, even more attractive QDs will be isolated that could gain approval for clinical, not just preclinical, purposes. For some biomedical applications, the longer acquisition times permitted by phosphorescent QDs such as ZnCuInS/ZnS may be advantageous. For example, imaging during the slow de-excitation phase when other confounding radiation such as scattered X-rays and shorter-lived autofluorescence has subsided, may result in increased image quality. Phosphorescent QDs could also be excited immediately *before* injecting into the biological sample, thus removing the need to expose the subject to X-rays. This contrasts with efforts to exploit phosphorescent crystals whose luminosity and decay timescales decrease as the crystal size is decreased below 1 micron^28^. Our finding of phosphorescence from nanometre-scale ZnCuInS/ZnS QDs offers a new potential target for bright phosphorescent NIR imaging of nanoparticles. Additionally, our detection of X-ray-induced optical radioluminescence from the natural biological tissue of the mouse presents a new challenge: to identify the biomolecules responsible, as the next step in understanding the nature, tissue-dependence and application of this phenomenon.

Looking to the future, the targeted delivery of QDs *in vivo* and *in vitro*, and their selective excitation by a more finely collimated X-ray beam, will allow higher resolution imaging than that demonstrated here, as well as allowing the emitted QD energy to be used selectively to trigger second-stage diagnostic or therapeutic actions. The penetrating power of X-rays offers the prospect of developing applications *in vivo* that are not confined to superficial organs or tissue sections. Such imaging, with good sensitivity and quick scan times, has the potential to contribute new biomedicine imaging armoury, as well as the ability to detect cellular and molecular processes in anatomical detail that could revolutionize non-invasive medical practise.

## Methods

### Quantum dots

As our interest was in biomedical imaging applications of QDs, we used QDs in aqueous suspension, rather than in dry form. Most of our work involved solid (powder) core-type CdTe QDs (10 mg, λ_emission_ = 715 nm; Sigma-Aldrich), functionalised with a carboxyl (COOH) group, dispersed in water. The QDs were shipped on ice, and stored refrigerated at ~4 °C except for the 2 hours or so on each occasion when suspensions were prepared or measurements made. A stock suspension in 2 ml of distilled water was created, and further diluted as required. The emission wavelength was verified using a Pasco PS-2600 benchtop spectrometer revealing peak λ_emission_ = 718 ± 2 nm and FWHM = 79 ± 3 nm. Phantoms were created as 0.1 ml volumes in 0.3 ml insulin syringes.

A second set of powdered QDs, core/shell ZnCuInS/ZnS (25 mg, λ_emission_ = 700 nm; Plasma-Chem) were procured, but being hydrophobic were dispersed in toluene. X-ray excitation of the ZnCuInS/ZnS QDs was confirmed.

### X-ray system theory

Clinical diagnostic X-ray machines generate X-rays extending from around 20 keV, limited at lower energy by the absorption of X-rays in the materials of the X-ray source, up to the (selectable) peak accelerating voltage (kV_p_) applied to the electron beam. Increasing the accelerating voltage not only extends the high-energy range of the X-rays, but (at fixed electron beam current) also increases the number (and dose) of X-rays produced at all energies; the intensity dependence is close to kV_p_^2^, though this exponent is not exact and depends on the filtration^29^. The spectrum in this range is dominated by a bremsstrahlung continuum, with additional tungsten Kα and Kβ radiations at 59 and 67 keV when kV_p_ exceeds these thresholds^29,30^.

For energies less than approximately 70–100 keV, the absorption of X-rays by CdTe is dominated by the photoelectric effect, for which the absorption coefficient decreases steeply with increasing energy, whereas Compton scattering dominates at higher energies. Cd and Te have K-shell ionisation edges at 26.7 keV and 31.8 keV respectively^24^, boosting the CdTe mass attenuation coefficient (*μ*/*ρ*) relative to water at energies just above these thresholds, though the mass energy-absorption coefficient (*μ*_en_/*ρ*) is raised less, suggesting little of the energy associated with the K-shell ionisation remains in the sample. The water mass energy-absorption coefficient is around a factor of 8 lower than the CdTe value.

### X-ray system methods

X-ray irradiation was undertaken using an APPELEM DaVinci Duo, with 3 mm Al filtration, housing a rotating tungsten anode and field-flattening filter. The collimator leaves were adjusted to produce a rectangular X-ray field appropriate to each sample. The machine was equipped with a DAP (Dose-Area Product) meter (accurate to +/− 10%), calibrated against a RaySafe dosimeter. It delivers a dose of 18 μGy/mAs at 55 kV_p_ for a source-to-subject distance of 1 m. Our typical exposure of 55 kV_p_ and 500 mAs at 0.5 m source-to-subject distance gives rise to a dose of 4 cGy. The beam current was decreased at 100 kV (Table 1) to partially compensate for the fact that increasing kV_p_ at fixed mA increases the X-ray flux at all energies.

### Mouse cadavers

Mouse cadavers were used for the purpose of demonstrating the transmission of X-rays and excitation of NIR emitting QDs in mammalian tissue. For Figs. 2 and 4(a,b), a white mouse cadaver (live weight “26 g plus”) with fur was obtained from the reptile pet-foodchain, previously euthanised and frozen. A second furred mouse from the same source was imaged in Fig. 4(c,d). For the deeper QD images giving rise to Fig. 3, a nude CD1 mouse was acquired from Charles River Laboratories and transported to UCL’s Centre for Advanced Biomedical Imaging for study. The mouse (26.5 g) was euthanised by Schedule 1 (overdose of anaesthesia: 5% isoflurane followed by 0.2 ml pentabarbitone sodium), then injected with 2 μl suspensions of CdTe (0.5 mg) at three locations: the left striatum, liver and left kidney. For the first, the head of the mouse cadaver was immobilised in a stereotaxic frame, and an injection was made using a 26 gauge 10 µl Hamilton syringe after drilling at bregma offsets AP = −0.2 mm, ML = +2 mm. The needle was advanced into the cerebral tissue (DV = −1.75 mm) and injection of 2 µl GdTe suspension performed over 5 mins. The needle was left *in situ* for a further 5 mins prior to it being removed. The liver and left kidney injections were made under ultrasound guidance (Visualsonics Vevo2100) to ensure correct deposition of the QDs, at measured depths 4 mm and 2 mm respectively, though these organ depths will change during repositioning due to the pliable nature of abdominal tissues. The CD1 mouse was imaged immediately after injections using an IVIS Lumina (Perkin Elmer) under DsRed excitation (500–550 nm) and Cy5.5 emission (690–770 nm), with a 1.57 s exposure time. A second nude mouse cadaver was imaged in Fig. 4(c,d). All live animal work was performed at UCL in accordance with the United Kingdom’s Animals (Scientific Procedures) Act of 1986 and was previously approved by UCL’s internal Animal Welfare and Ethical Review Body.

### NIR imaging

Our bespoke imaging system consisted of a light-tight housing with the X-ray source overhead, at a source-to-subject distance of 50 cm. We used a Hamamatsu C9100-23B 512 × 512 pixel EM-CCD detector (quantum efficiency ~88% at ~715 nm), operating at −65 °C and positioned at 90° to the X-ray beam. Based on a silicon semiconductor, it has no sensitivity to the thermal infrared (or other wavelengths > 1100 nm). It was fitted with an unfiltered 35 mm focal length, *f*/1.4 lens (Nikon Nikkor) for Fig. 1, and a 28 mm focal-length, *f*/1.4 NIR lens (Edmund Optics) with a narrow bandpass filter (λ_c_ = 700 nm, FWHM = 70 nm; 700FIW25, Knight Optical) for Figs. 2 and 3, positioned 30 cm from the sample, out of the incident X-ray beam. For Fig. 4, 575FCS2500 = BG39 (320–660 nm) and 665FCS2500 = RG665 (λ > 660 nm) filters (Knight Optical) were used. Narrower-band filters 550FIW25, 450FIW25, and 400FIR25 (Knight Optical) were also used to inspect the endogenous emission (images not shown). The EM-CCD is also sensitive to X-rays scattered out of the main X-ray beam, so 1.8 mm lead shielding was added to the light-tight box for the images that constitute Figs. 2 and 3, and 2.4 mm shielding was used for Fig. 4. Even so, a small number of scattered X-ray “hits” are visible as point-like bright spots in these figures. The EM-CCD sensitivity gain was set at 180 (38 ADU per detected photon) for the syringes in Fig. 1, and 200 (56 ADU per photon) for Figs. 2, 3 and 4.

### Image processing

Image processing and analysis was undertaken using ImageJ^31^. To reduce the impact of scattered X-rays on image statistics, a median filter with a radius of 3–5 pixels was applied prior to the analysis of Fig. 1, since in the presence of anomalously high counts due to scattered X-ray hits, the median provides a more robust central-value estimator than the mean. The use of lead shielding when acquiring Figs. 2, 3 and 4 reduced the need for median filtering in those images; a median filter with radius = 1 pixel was applied to Figs. 3 and 4 to reduce noise. In most cases, three successive images of X-ray-excited QD emission were taken for each view, and a maximum-pixel rejection algorithm was applied to reject the brightest occurrence in each three-frame series for each pixel.

### Operational efficiency of X-ray-to-NIR energy conversion

We define the operational efficiency of the QDs as the NIR energy radiated into 4π steradians divided by the X-ray energy absorbed (by both CdTe and H_2_O) in the suspension. We adopt the mass-energy absorption coefficients at the mean photon energy of the beam, obtained from a TASMICS calculation^30^, rather than by computing the absorption differentially with energy throughout the spectrum; this estimate could be refined in future work, but for only marginal benefit. The NIR emission is measured from the images using the known incident photon-to-ADU gain factor and the quantum efficiency of the CCD. The sample-to-camera distance and aperture of the camera lens are used to calculate the emitted photon count over 4π steradians, assuming the QDs emit isotropically.

## Data Availability

The raw data underpinning Figs. 1–4, and in addition two stop-motion video sequences incorporating Figs. 2a and 3a, will be made available upon publication at the following URL: http://researchprofiles.herts.ac.uk/portal/en/persons/sean-ryan(978f1ae5-d0db-42ec-a3cc-fdb8e553429e)/publications.html
